# Development of a Mast‐Cell Activation Test for Diagnosing Allergic Rhinitis

**DOI:** 10.1155/jimr/6317442

**Published:** 2026-11-09

**Authors:** Jianli Pan, Ling Gong, Jie Xu, Shiqiong Luo, Chao Wang, Rui Liu, Tao Zhang, Ming Zhang

**Affiliations:** ^1^ Department of Respiratory and Critical Care Medicine The Second Affiliated Hospital of Xi’an Jiaotong University Xi’an Shaanxi China xjtu.edu.cn; ^2^ Department of Special Need Xi’an Children’s Hospital Xi’an Shaanxi China xachyy.com; ^3^ School of Pharmacy Xi’an Jiaotong University Xi’an 710061 Shaanxi Province China xjtu.edu.cn

**Keywords:** allergic rhinitis, CD63, flow cytometry, mast cells, serum

## Abstract

Serum‐specific IgE (sIgE) and skin prick testing (SPT) have limitations in diagnosing allergic rhinitis (AR). CD63, a mast cell activation biomarker reflecting degranulation extent, has unclear application in AR diagnosis. This study developed a functional mast cell activation test (MAT) for the in vitro diagnosis of AR by quantifying CD63 expression on LAD2 cells stimulated with patient serum and specific allergens. The assay was evaluated in 128 AR patients and 115 controls. The assay showed good reproducibility (CV = 8.26%). In the test cohort, AR patients had significantly higher CD63‐positive rates than controls (5.21% ± 4.55% vs. 1.39% ± 1.79%, *p* < 0.001). ROC analysis yielded an AUC of 0.84, with 94.60% specificity and 77.10% accuracy at a 2.09% cutoff, which was confirmed in the validation cohort (specificity 90.24%, accuracy 80.51%). In the overall population, CD63 AUC (0.85) was higher than that for histamine (0.68) and total IgE (tIgE, 0.83). The CD63‐positive rate correlated strongly with sIgE (*r* = 0.59, *p* < 0.001) and total IgE (*r* = 0.42, *p* < 0.001), whereas its correlation with histamine release was weaker (*r* = 0.35 vs. sIgE). These findings support MAT based on mast cell CD63 as a precise functional tool for AR diagnosis.

## 1. Introduction

Allergic rhinitis (AR) is a globally prevalent, IgE‐mediated inflammatory disease of the nasal mucosa, characterized by symptoms such as sneezing, rhinorrhea, and nasal congestion [1]. Its significant and rising prevalence, particularly in urbanized areas, coupled with its detrimental impact on sleep, cognitive function, and quality of life, underscores the necessity for accurate diagnosis and effective management [2–4].

Current diagnostic practice for AR involves the evaluation of clinical symptoms and confirmation through allergen testing [5]. Although valued for its simplicity and low cost, the skin prick test (SPT) has limitations, including unsuitability for patients with extensive dermatoses and the potential to provoke systemic reactions [6, 7]. In vitro, the detection of serum‐specific IgE (sIgE) is highly specific for confirming sensitization to particular allergens. However, a positive sIgE result merely indicates immunologic sensitization and does not necessarily correlate with the clinical severity of the allergic response. Other methods, such as the basophil activation test (BAT), have emerged as functional cellular assays that bridge this gap by measuring the activation of key effector cells [8]. BAT demonstrates superior specificity and sensitivity compared to sIgE detection. Nevertheless, its widespread clinical adoption is hampered by several practical limitations: it requires fresh whole blood samples, involves a technically challenging and time‐consuming isolation of basophils (which are rare in circulation) [9], and exhibits a notable rate of nonresponders (~10%–15%), leading to potential false negatives [10].

To address the limitations of current diagnostic methods such as BAT, we developed a novel mast cell activation test (MAT). Mast cells are pivotal effector cells in allergic inflammation, and their activation and subsequent degranulation directly underlie the clinical manifestations of AR. The principle of MAT is to simulate the in vivo sensitization process in vitro; the human mast cell line LAD2 is first sensitized by incubation with patient serum containing specific IgE [11]. Following this, the cells are challenged with relevant allergens, and the degree of mast cell activation is quantitatively measured via flow cytometric detection of CD63, a tetraspanin protein that translocates from the membranes of secretory granules to the cell surface upon degranulation [12]. This surface expression of CD63 thus serves as a robust functional biomarker of mast cell activation.

In this study, we established a standardized MAT protocol and evaluated its diagnostic performance in a cohort of well‐characterized AR patients and healthy controls. By circumventing the need for fresh blood samples and complex cell isolation procedures—as required in the BAT—MAT improves reproducibility and practical utility. Furthermore, we compared MAT against established diagnostic metrics, including serum sIgE, total IgE (tIgE), and histamine release, to assess its potential as a superior functional tool for in vitro allergy diagnosis.

## 2. Materials and Methods

### 2.1. Reagents and Cell Line

Key reagents were sourced as follows: human SCF, StemPro‐34 nutrient supplement and medium (Invitrogen, USA), human IgE (BioPorto, Denmark), streptavidin (MedChemExpress, USA), CD63‐PE antibody, Human TruStain FcX (BioLegend, USA), 7‐AAD‐PerCP (BD Biosciences, USA), *Artemisia annua* allergen extract (Zhejiang Wolw, China), β‐hexosaminidase (Macklin, China), modified Tyrode’s buffer, DMSO (Aladdin, China), Triton X‐100 lysis buffer (Kehao, China), D‐PBS (Coolaber, China), and BSA (Pioneer Biotech, China).

The human mast cell line LAD2 (male, bone marrow; RRID: CVCL_0387) was obtained from Zhenjiang abm Biotechnology Co., Ltd. (abm, China; Catalog Number T8157) on May 29, 2023. STR authentication (21‐STR, ABI3730XL) was performed. Because LAD2 STR data are not archived in public databases (ATCC/DSMZ/JCRB/RIKEN), no percentage match could be calculated. However, the profile matched an in‐house reference established by abm through cumulative analyses of multiple batches and cross‐verified against their internal LAD2 controls, with no multiallelic events, confirming cell identity. This cell line has not been reported as misidentified or contaminated, and it tested negative for mycoplasma by PCR. Cells were cultured at 37°C in a humidified atmosphere of 5% CO_2_ using StemPro‐34 SFM medium supplemented with 100 ng/mL SCF, 100 U/mL penicillin‐streptomycin, 2 mmol/L L‐glutamine, and nutrient supplement. Cells were maintained by semimedium exchange and passaged when the density exceeded 5 × 10^5^ cells/mL. According to the supplier’s information (abm Biotechnology Co., Ltd., China), LAD2 cells typically exhibit a doubling time of ~2–4 weeks under optimal culture conditions.

### 2.2. MAT Development and Optimization

LAD2 cells were sensitized with biotinylated human IgE (1 μg/mL) and activated by streptavidin (0.25–1 μg/mL for 30 min)‐mediated FcεRI crosslinking as a standardized positive control for evaluating mast cell responsiveness. Activation was assessed via β‐hexosaminidase release (colorimetric assay), histamine (LC‐MS/MS), and CD63 surface expression (flow cytometry). The optimal stimulation duration (15–60 min at 1 μg/mL) was similarly evaluated. To assess IL‐33 priming, cells were pretreated with 100 ng/mL IL‐33 for 20 min following sensitization and then stimulated with streptavidin.

For flow cytometry staining optimization, sensitized and IL‐33‐primed cells were activated with 1 μg/mL streptavidin, blocked with Human TruStain FcX, and stained with serial dilutions of CD63‐PE (0.157–2.500 μg/mL; 30 min, 4°C) and 7‐AAD‐PerCP (0.313–5 μg/mL; 5 min). Optimal staining duration was determined using CD63‐PE (0.625 μg/mL, 30–60 min) and 7‐AAD‐PerCP (1.250 μg/mL, 5–50 min). Stained cells were analyzed by flow cytometry to define optimal conditions based on the signal‐to‐noise ratio.

### 2.3. MAT Precision Assessment

Precision was evaluated across three independent batches. LAD2 cells were sensitized, primed with IL‐33 (100 ng/mL, 20 min), and activated with 1 μg/mL streptavidin (30 min). After blocking, cells were stained with CD63‐PE (0.625 μg/mL, 60 min) and 7‐AAD‐PerCP (1.25 μg/mL, 5 min). CD63‐positive rates were determined by flow cytometry, and interbatch coefficient of variation (CV) was calculated.

### 2.4. MAT Applicability and Specificity Assessment

The cytotoxicity of *A. annua* allergen extract (33–3300 DU/mL) was assessed by the CCK‐8 assay. For applicability, LAD2 cells sensitized with patient serum were primed with IL‐33 and challenged with allergen (0–660 DU/mL); activation was measured via CD63 expression. For specificity, sera from four groups (Artemisia‐allergic, dust mite‐allergic, dual‐sensitized, and healthy controls) were used for sensitization. Cells were challenged with 660 DU/mL Artemisia extract, with negative (Tyrode’s buffer) and positive (1 μg/mL streptavidin) controls, and analyzed by flow cytometry.

### 2.5. Study Population and Sample Collection

The study was approved by the Medical Ethics Committee (Approval Number 2024035). Inclusion criteria for the case group were age 18 − 65 years, clinically confirmed AR with sIgE to weed pollen ≥ class 2 or SPT ≥ ++, and no sensitization to dust mites, molds, or pets. Controls were age‐matched healthy volunteers without allergic symptoms. Exclusion criteria included malignancy, pregnancy, lactation, or significant comorbidities. Medication history was recorded for all participants, including the use of oral or topical antihistamines, oral corticosteroids, and intranasal corticosteroids. None of the participants had received allergen immunotherapy (AIT) or biological agents targeting IgE or other immune pathways before enrollment. Additionally, the use of traditional Chinese medicines, herbal products, or supplements was recorded; only one AR patient reported the use of traditional Chinese medicine.

From July 2024 to July 2025, 243 subjects were enrolled at Yulin Hospital (Xi’an Jiaotong University). After random allocation, the case group comprised 128 Artemisia‐sensitized AR patients (58 male, 70 female, mean age 29.60 ± 15.50 years), and the control group comprised 115 healthy individuals (37 male, 78 female, mean age 39.20 ± 12.70 years). Demographics, family history, medication use, and symptom scores (TNSS and VAS) were recorded. Venous blood (6 mL) was collected, clotted at room temperature for ≥60 min, centrifuged at 3000 rpm for 20 min, and serum aliquots stored at −80°C. tIgE and sIgE were measured by ELISA.

### 2.6. MAT and Histamine Analysis in Clinical Samples

For MAT, LAD2 cells were sensitized with participant serum, primed with IL‐33, and stimulated with 660 DU/mL Artemisia extract. Activation was quantified by CD63‐PE staining and viability by 7‐AAD‐PerCP via flow cytometry.

Histamine release was measured by LC‐MS/MS. After stimulation (negative control, positive control with 1 μg/mL streptavidin, or 660 DU/mL allergen), 50 μL supernatant was mixed with 100 μL histamine internal standard in acetonitrile, vortexed, centrifuged, filtered (0.22 μm), and analyzed.

### 2.7. Statistical Analysis

Data were analyzed with GraphPad Prism 8.3.0 and FlowJo 10.8.1. Comparisons across groups used one‐way ANOVA. Continuous data are expressed as the mean ± SEM. Statistical significance was set at *p*  < 0.05.

## 3. Results

### 3.1. Establishment of an IgE‐Dependent MAT Assay

Crosslinking of IgE bound to the high‐affinity receptor FcεRI triggers mast cell degranulation. To evaluate activation, we measured three established degranulation markers, β‐hexosaminidase (β‐Hex), histamine, and surface CD63. Streptavidin stimulation induced concentration‐dependent β‐Hex release, increasing from 20.98% ± 0.57% at 0.25 µg/mL to 35.59% ± 1.11% at 1 µg/mL, compared to 16.22% ± 0.64% in the vehicle group (Figure 1A–A1). Histamine release showed a similar trend, rising from 51.58 ± 0.95 ng/mL (vehicle) to 139.07 ± 13.93 ng/mL at 1 µg/mL streptavidin (Figure 1A–A2). Time‐course analysis at 1 µg/mL revealed peak responses at 30 min, β‐Hex release reached 55.75% ± 1.36% (Figure 1B–B1), and histamine release was 103.90 ± 2.27 ng/mL (Figure 1B–B2). Flow cytometry confirmed concentration‐dependent CD63 surface expression (Figure 1C), with 85.75% ± 1.25% positive cells at 1 µg/mL versus 22.00% ± 0.20% and 36.50% ± 5.30% at 0.25 and 0.5 µg/mL, respectively (Figure 1D–D1). The complete gating strategy for flow cytometry analysis is shown in Figure S1. CD63‐positive cells accounted for 74.85% ± 0.55% when incubated with the antibody for 30 min (Figure 1D–D2), and shorter or longer incubations yielded lower values. These findings establish that 1 µg/mL streptavidin applied for 30 min robustly activates IgE‐sensitized mast cells, providing a reliable model for subsequent studies.

**Figure 1:**
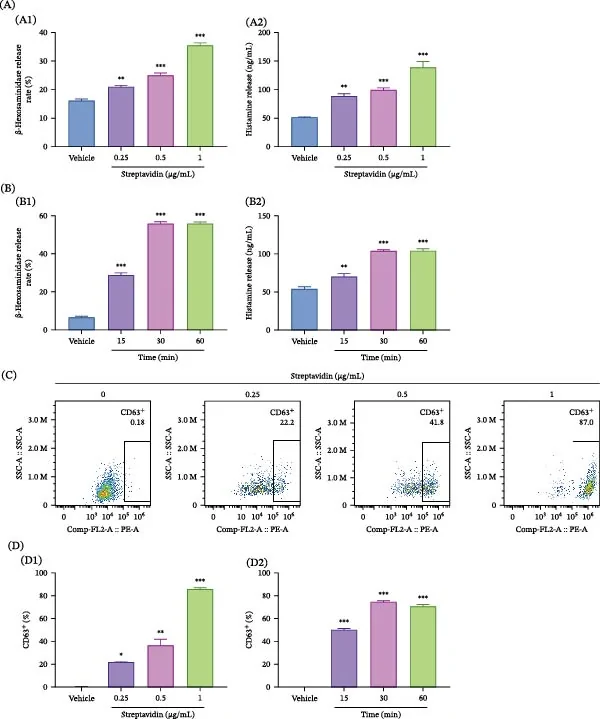
Dose‐ and time‐dependent effects of streptavidin‐induced mast cell degranulation and dynamic regulation of CD63 expression by streptavidin. (A) Release of β‐Hex (A1) and histamine (A2) from mast cells stimulated with increasing concentrations of streptavidin. (B) Release of β‐Hex (B1) and histamine (B2) from mast cells stimulated with 1 μg/mL streptavidin for varying durations. (C) Representative flow cytometry plots showing CD63^+^ mast cell populations after stimulation with graded streptavidin concentrations. (D) Quantitative analysis of CD63^+^ cell percentages following stimulation with increasing streptavidin concentrations (D1), or after stimulation with 1 μg/mL streptavidin over time (D2). Data are presented as mean ± SEM (*n* = 3).  ^∗^ *p* < 0.05,  ^∗∗^ *p* < 0.01, and  ^∗∗∗^ *p* < 0.001 versus vehicle control by one‐way ANOVA with Dunnett’s post hoc test.

### 3.2. IL‐33 Priming Enhances Streptavidin‐Induced Mast‐Cell Degranulation

IL‐33 is an alarm protein derived from epithelial cells and is involved in type 2 allergic inflammation [13]. By enhancing the reactivity of mast cells through the ST2 signaling pathway, IL‐33 can amplify the weak IgE/FcεRI‐mediated activation [14]. Therefore, including IL‐33 pretreatment in the MAT experimental protocol is necessary to simulate the allergic inflammatory environment and increase the activation sensitivity of mast cells. As shown in Figure 2, pretreatment with 100 ng/mL IL‐33 markedly enhanced mast cell activation under subthreshold streptavidin stimulation (0.25 µg/mL). β‐Hex release rose from 20.98% ± 0.57% to 26.58% ± 0.49% (*p* < 0.001, Figure 2A), histamine secretion increased from 88.67 ± 5.64 ng/mL to 115.93 ± 3.68 ng/mL (*p* < 0.001, Figure 2B), and the CD63‐positive cell percentage increased from 21.00% ± 0.20% to 58.00% ± 0.35% (*p* < 0.001, Figure 2C). These results indicate that IL‐33 amplifies IgE/FcεRI‐mediated activation by presensitizing the intracellular signaling network, thereby enhancing cellular responsiveness to low‐intensity allergen challenge. Notably, IL‐33 priming alone did not directly induce degranulation but rendered mast cells hyperresponsive (primed but not triggered), enabling them to exceed the conventional activation threshold upon subthreshold stimulation and initiate signal amplification. These findings demonstrate that IL‐33 priming enhances CD63 responses to subthreshold IgE/FcεRI‐mediated stimulation, supporting its use as a signal‐amplification step in the optimized MAT protocol.

**Figure 2:**
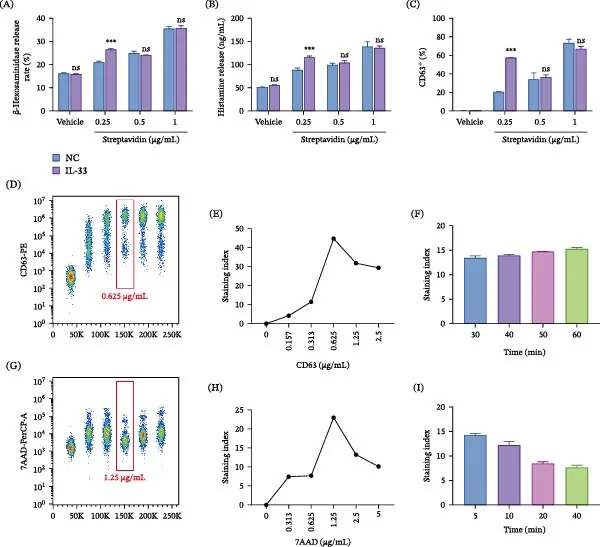
Enhanced mast cell activation by IL‐33 priming and optimization of flow cytometry staining conditions. (A–C) IL‐33 priming enhances streptavidin‐induced LAD2 mast cell activation. Quantification of (A) β‐hexosaminidase (β‐Hex) release, (B) histamine release, and (C) flow cytometric analysis of CD63^+^ cell percentages (compared with vehicle control group,  ^∗∗∗^ *p* < 0.001, *n* = 3). (D) Separation of negative and positive mast cell populations stained with graded concentrations of PE‐CD63 antibody. (E) Staining index (SI) calculated based on fluorescence intensity from (D). (F) SI values for CD63‐PE at different incubation times. (G) Separation of negative and positive mast cell populations stained with graded concentrations of 7AAD‐PerCP‐A antibody. (H) SI calculated based on fluorescence intensity from (G). (I) SI values for 7AAD‐PerCP‐A antibody at different incubation times. Data are presented as mean ± SEM (*n* = 3).  ^∗∗∗^ *p* < 0.001 versus the NC‐IL‐33 group by a two‐way ANOVA followed by Šidák’s multiple comparisons test; ns, not significant.

### 3.3. Optimization of Antibody Staining Conditions for Flow Cytometry

To establish robust flow cytometric detection, we optimized the staining concentrations and incubation times for CD63‐PE and 7‐AAD‐PerCP. As shown in Figure 2D,E, CD63‐PE at concentrations below 0.625 µg/mL resulted in poor separation between positive and negative populations and a low staining index. A concentration of 0.625 µg/mL significantly improved population resolution and the staining index, whereas higher concentrations reduced performance. We next evaluated the effect of the staining duration. Extending CD63‐PE incubation from 30 to 60 min progressively increased the staining index from 13.45 to 15.30 (Figure 2F). Similarly, for 7‐AAD‐PerCP, a concentration of 1.250 µg/mL provided optimal separation and staining index, with inferior results at both lower and higher concentrations (Figure 2G,H). In contrast, prolonging 7‐AAD‐PerCP staining beyond 5 min reduced the staining index, with values declining from 14.30 at 5 min to 7.66 at 40 min (Figure 2I). These results establish that staining with 0.625 µg/mL CD63‐PE for 60 min and 1.250 µg/mL 7‐AAD‐PerCP for 5 min optimally enhances population separation and staining index, providing a validated staining protocol for subsequent mast cell activation assays.

### 3.4. MAT Demonstrates Good Interbatch Precision

The precision of the MAT was assessed across three independent batches by measuring the variation in the CD63‐positive cell rate induced by standard stimulation. The results (85.75%, 77.30%, and 70.05%) showed a CV of 8.26%, which is below the preset acceptance threshold (CV ≤ 15%). To further assess interbatch precision and functional consistency of the MAT, baseline and positive‐control CD63 responses were evaluated across five independent experimental batches (Table S1). This confirms that the MAT has satisfactory interbatch precision and stable reproducibility, meeting the requirements for reliable practical use.

### 3.5. Applicability Assessment of the MAT

The CCK‐8 assay showed that *A. annua* allergen extract did not affect LAD2 cell viability at concentrations up to 660 DU/mL (*p* > 0.05 vs. control), whereas a significant reduction occurred at 3300 DU/mL (*p* < 0.01, Figure 3A). Therefore, a concentration range of 0–660 DU/mL was selected for subsequent experiments. To determine the optimal allergen concentration for MAT, we performed dose‐response experiments using sera from *A. annua*‐allergic patients. CD63 levels increased in a concentration‐dependent manner upon *A. annua* allergen stimulation, peaking at 660 DU/mL (11.12% ± 2.74%), which was significantly higher than levels at 33 DU/mL (5.51% ± 2.62%) and 330 DU/mL (8.04% ± 2.47%) (Figure 3B,C). Based on these optimization results, 660 DU/mL was selected as the standardized concentration for subsequent clinical testing to ensure assay consistency, reproducibility, and reliable cutoff determination.

**Figure 3:**
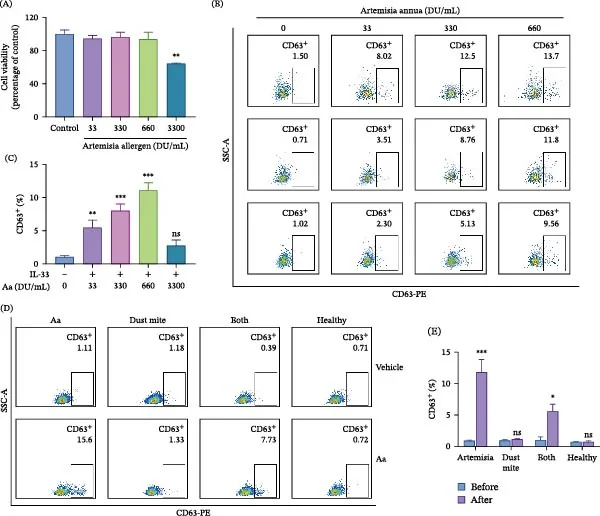
Effects of *Artemisia annua* allergen on mast cell viability and CD63^+^ expression, and impact of serum sensitization on allergen‐induced CD63^+^ expression. (A) Viability of mast cells exposed to *Artemisia annua* allergen. (B) Representative flow cytometry plots showing CD63^+^ expression patterns. (C) Quantitative analysis of CD63^+^ cell percentages after stimulation with graded concentrations of *Artemisia annua* allergen. (D) Representative flow cytometry plots showing CD63^+^ expression patterns in mast cells sensitized with distinct sera and stimulated with *Artemisia* allergen (Aa). (E) Quantitative analysis of CD63^+^ cell percentages among groups sensitized with different sera after Aa stimulation (compared with vehicle control group,  ^∗∗^ *p* < 0.01,  ^∗∗∗^ *p* < 0.001, and *n* = 3). Data are presented as mean ± SEM (*n* = 3).  ^∗∗^ *p* < 0.01,  ^∗∗∗^ *p* < 0.001 versus vehicle control by one‐way ANOVA with Dunnett’s post hoc test (A–C). Two‐way ANOVA followed by Šidák’s multiple comparisons test was used to compare CD63^+^ expression before and after stimulation within each group (E).  ^∗^ *p* < 0.05,  ^∗∗∗^ *p* < 0.001; ns, not significant.

To evaluate the specificity of the MAT, we measured CD63 expression on mast cells sensitized with sera from different donor groups following *A. annua* allergen challenge. As shown in Figure 3D,E, a marked increase in CD63 expression was observed only in cells sensitized with serum from *A. annua*‐allergic patients (11.82% ± 3.47%). In contrast, cells sensitized with serum from dust mite‐allergic individuals or healthy controls showed negligible activation. The serum from patients allergic to both *A. annua* and dust mite induced an intermediate response (5.57% ± 1.97%). These results demonstrate that the MAT specifically detects *A. annua*‐specific IgE‐mediated activation, without cross‐reactivity to dust mite‐specific IgE or nonspecific serum factors, confirming its suitability for clinical diagnosis.

### 3.6. Baseline Clinical Characteristics of the Study Cohort

A total of 243 subjects were included, comprising 128 *A. annua*‐sensitized AR patients and 115 healthy controls. The case group had a mean age of 29.60 ± 15.50 years and a mean disease duration of 9.04 ± 5.51 years, with 58 males (45.3%) and 70 females (54.7%). Age stratification showed a higher proportion of males in the <20 years subgroup, while females predominated in the 20–40 and 40–60 years subgroups. The gender distribution differed significantly between the case and control groups (*p* < 0.05). Immunological profiling revealed markedly elevated tIgE levels in the case group (298.80 ± 28.91 IU/mL) compared to the control group (93.07 ± 71.90 IU/mL, *p*  < 0.001). Similarly, *A. annua*‐specific IgE levels were significantly higher in AR patients (49.78 ± 47.12 IU/mL, *p*  < 0.001) (Figure 4A,B, Table 1). In summary, the case group exhibited a distinct allergic immune phenotype characterized by significantly elevated total and allergen‐specific IgE levels.

**Figure 4:**
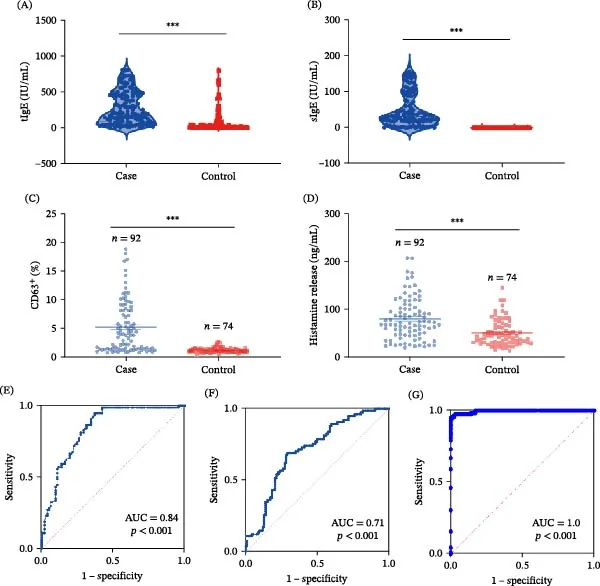
Immunological profiles, mast cell activation, and diagnostic performance of biomarkers in allergic rhinitis. (A) Serum total IgE levels in AR cases versus healthy controls (case: *n* = 128; control: *n* = 115; and  ^∗∗∗^ *p* < 0.001 vs. control group). (B) Allergen‐specific IgE levels (case: *n* = 128; control: *n* = 115; and  ^∗∗∗^ *p* < 0.001 vs. control group). (C) CD63^+^ expression levels in allergen‐stimulated mast cells from AR cases versus healthy controls (case: *n* = 92; control: *n* = 74; and  ^∗∗∗^ *p* < 0.001 vs. control group). (D) Histamine release from allergen‐stimulated mast cells in AR cases and controls (case: *n* = 92; control: *n* = 74; and  ^∗∗∗^ *p* < 0.001 vs. control group). (E) ROC curve of CD63^+^ expression (test set, *n* = 166). (F) ROC curve of histamine release (test set, *n* = 166). (G) ROC curve of allergen‐specific IgE (sIgE) (case: *n* = 128; control: *n* = 115; and AUC = 1.0,  ^∗∗∗^ *p* < 0.001). Data are presented as mean ± SEM (*n* = 3). Statistical significance was determined by the unpaired *t*‐test for two‐group comparisons.

**Table 1:** Demographic data.

Characteristic	Case group (*n* = 128)	Control group (*n* = 115)
Number of subjects	128	115
Sex, *n* (%)	—	—
Male	58 (45.3%)	37 (32.2%)
Female	70 (54.7%)	78 (67.8%)
Age, years	29.60 ± 15.50	39.20 ± 12.70
Disease duration, years	9.04 ± 5.51	NA
VAS score	7.50 ± 2.37	NA
TNSS score	9.47 ± 2.76	NA
Total IgE, IU/mL	298.8 ± 28.91	93.07 ± 71.90
Specific IgE, IU/mL	49.78 ± 47.12	0.21 ± 0.76

*Note:* Data are presented as mean ± standard deviation.

### 3.7. MAT Detects Differential Mast‐Cell Activation Between Mugwort‐Allergic Patients and Healthy Controls

The 243 samples were randomly divided into a test set (*n* = 166) and a validation set (*n* = 77) in a 7:3 ratio. Using the MAT, mast cells sensitized with serum from *A. annua*‐allergic patients (*n* = 92) showed significantly higher activation than those sensitized with serum from healthy controls (*n* = 74). Flow cytometric analysis revealed that the CD63‐positive rate was 5.21% ± 4.55% in the case group versus 1.39% ± 1.79% in the control group (Figure 4C), confirming specific activation of the IgE/FcεRI pathway in allergic individuals. Consistent with this, histamine release—assayed by LC‐MS/MS—was also significantly elevated in the case group (81.21 ± 43.64 ng/mL) compared to controls (52.00 ± 27.64 ng/mL, Figure 4D), supporting the reliability of CD63 as a degranulation marker. To evaluate whether systemic corticosteroid exposure influenced MAT performance, a sensitivity analysis was performed after excluding all participants with oral corticosteroid use. After exclusion, CD63‐positive rates and histamine release remained significantly higher in AR patients than in healthy controls (both *p*  < 0.001, Figure S2). Univariate logistic regression further demonstrated that both CD63 and histamine release were significantly associated with AR. The CD63‐positive rate showed a strong association with disease risk (*B* = 0.67, *p*  < 0.01), with an odds ratio (OR) of 1.96, indicating that each 1% increase in CD63 expression was associated with a 96% rise in the odds of having AR. In contrast, although histamine release was also statistically significant (*B* = 0.22, *p*  < 0.01), its effect size was modest (OR = 1.02), corresponding to only a 1.9% increase in risk per unit change. Thus, CD63 expression effectively distinguishes *A. annua*‐sensitized AR patients from healthy controls and demonstrates stronger diagnostic relevance than histamine, supporting its use as a primary functional biomarker in the MAT, with histamine serving as a supplementary indicator of mast cell degranulation.

### 3.8. Diagnostic Performance of the MAT Based on ROC Curve Analysis

We generated ROC curves to evaluate the diagnostic utility of CD63‐positive rate and histamine release and allergen‐specific IgE (sIgE) for AR. As shown in Figure 4E–G, the AUC for CD63 was 0.84. The optimal cutoff determined by the maximum Youden index was 2.09%, representing the absolute percentage of CD63‐positive cells after allergen stimulation. At this cutoff, CD63 detection achieved a specificity of 94.60%, a sensitivity of 62.70%, and an overall accuracy of 77.10%. This performance was superior to that of histamine release, which yielded an AUC of 0.71, with a specificity of 68.90% and an accuracy of 70.50% at a cutoff of 56.15 ng/mL. In addition, sIgE yielded an AUC of 1.0 (*p* < 0.001; Figure 4G). However, this near‐perfect discrimination should be interpreted with caution as positive sIgE was an inclusion criterion for the AR group. Thus, ROC analysis indicates that MAT based on CD63 detection exhibits high specificity and favorable diagnostic performance for AR, outperforming histamine release as a discriminative biomarker.

### 3.9. Validation of the MAT Diagnostic Model and Performance Evaluation

The optimal cutoff values, determined by maximizing the Youden index in the test set, were 2.09% for CD63 and 56.15 ng/mL for histamine release. These cutoff values were subsequently applied to the independent validation set (n = 77) to evaluate diagnostic performance. As shown in Figure 5A, CD63 detection maintained high performance with a specificity of 90.24% and an accuracy of 80.51% (AUC = 0.88), producing only 4 false‐positive results. In contrast, histamine detection showed markedly declined performance in the validation set, with specificity dropping to 58.54%, accuracy to 62.34% (AUC = 0.60) and false positives increasing to 17 cases (Figure 5B). A comparative analysis of diagnostic metrics between the test and validation sets (Table 2) revealed that CD63 consistently achieved high AUC values (0.84 and 0.88, respectively), with slight improvements in sensitivity and specificity in the validation set. This indicates robust generalizability and stable performance across datasets. Conversely, histamine exhibited lower and inconsistent AUC values (0.71 and 0.60), with decreased sensitivity, specificity, and accuracy upon validation, underscoring its limitations as a standalone diagnostic marker. Therefore, the MAT based on CD63 detection demonstrates stable and reliable diagnostic performance across independent datasets, supporting its potential utility as an auxiliary in vitro diagnostic tool for AR.

**Figure 5:**
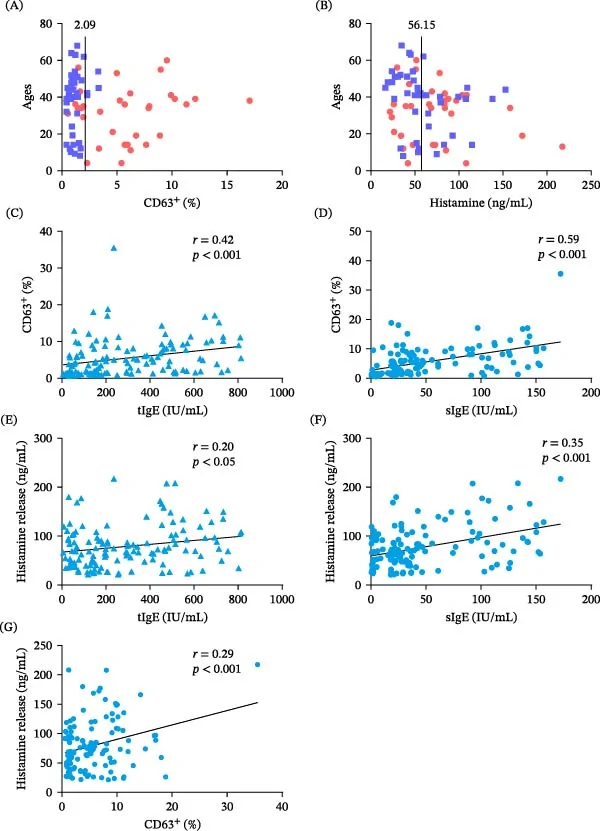
Validation and correlation analysis of MAT performance. (A) CD63^+^ expression levels in the validation cohort (healthy controls in blue, *Artemisia annua*‐allergic patients in red; *n* = 77). (B) Histamine release levels in the validation cohort (*n* = 77). (C) Spearman’s correlation analysis of CD63^+^ expression with total IgE (tIgE) levels (*r* = 0.42,  ^∗∗∗^ *p* < 0.001, and *n* = 128). (D) Spearman’s correlation analysis of CD63^+^ expression with allergen‐specific IgE (sIgE) levels (*r* = 0.59,  ^∗∗∗^ *p* < 0.001, and *n* = 128). (E) Spearman’s correlation analysis of histamine release with tIgE levels (*r* = 0.20, *p*  < 0.05, and *n* = 128). (F) Spearman’s correlation analysis of histamine release with sIgE levels. (G) Spearman’s correlation analysis of CD63^+^ expression with histamine release (*r* = 0.29,  ^∗∗∗^ *p* < 0.001, and *n* = 128). Data points represent individual measurements (*r* = 0.35,  ^∗∗∗^ *p* < 0.001, and *n* = 128).

**Table 2:** Comparison of MAT test and validation sets.

Variables	Test set		Validation set	
	CD63	Histamine	CD63	Histamine
Number of subjects	166	166	77	77
AUC	0.84	0.71	0.88	0.60
Sensitivity, %	62.70	71.70	69.44	66.67
Specificity, %	94.60	68.90	90.24	58.54
Accuracy, %	77.10	70.50	80.51	62.34
Positive predictive value, %	93.50	74.20	86.21	58.54
Negative predictive value, %	67.30	66.20	77.08	66.67
Positive likelihood ratio	11.61	2.30	7.11	1.61
Negative likelihood ratio	0.39	0.41	0.33	0.57
False positive rate, %	5.40	31.10	9.76	41.46
False negative rate, %	37.30	28.30	30.56	33.33
Youden’s index	0.57	0.41	0.60	0.25

### 3.10. Comparative Analysis of MAT Diagnostic Performance

We compared the diagnostic performance of MAT (CD63 detection) with tIgE and histamine release using ROC analysis. As summarized in Table 3, CD63 demonstrated an AUC of 0.85, with 65.0% sensitivity, 93.0% specificity, 91.7% PPV, 72.8% NPV, 78.2% accuracy, and a Youden’s index of 0.58. These results indicate strong diagnostic efficacy, particularly in terms of specificity. tIgE showed comparable performance (AUC = 0.83), with higher sensitivity (73.0%) but lower specificity (83.6%), resulting in a Youden’s index of 0.57. In contrast, histamine release exhibited limited diagnostic value (AUC = 0.68), with 66.0% sensitivity, 57.0% specificity, and a Youden’s index of 0.23. Thus, MAT based on CD63 detection outperforms both histamine release and serum tIgE in terms of AUC, specificity, and overall diagnostic accuracy. Its high specificity and low false‐positive rate make it a valuable auxiliary diagnostic tool for *A. annua* pollen‐induced AR.

**Table 3:** ROC curve output results (full cohort comparison).

Variables	CD63+	Histamine	Total IgE
Sample size	243	243	243
AUC	0.85	0.68	0.83
Specificity, %	93.00	57.00	83.60
Sensitivity, %	65.00	66.00	73.00
Positive predictive value, %	91.70	64.90	77.40
Negative predictive value, %	72.80	57.60	79.20
Accuracy, %	78.20	61.70	78.00
Youden’s index	0.58	0.23	0.57

### 3.11. Correlation Between MAT Markers and Established Clinical IgE Parameters

Spearman’s correlation was used to evaluate the association between MAT‐derived markers and routine clinical IgE assays. As shown in Figure 5C,D, CD63‐positive rate showed a moderate correlation with tIgE levels (*r* = 0.42, *p* < 0.001) and a strong positive correlation with sIgE levels (*r* = 0.59, *p* < 0.001). In contrast, histamine release was only weakly correlated with tIgE (*r* = 0.20, *p* < 0.05) and sIgE (*r* = 0.35, *p* < 0.001) (Figure 5E,F). Furthermore, a significant positive correlation was observed between CD63‐positive rate and histamine release (*r* = 0.29, *p*  < 0.001; Figure 5G). These findings indicate that CD63 expression, as detected by MAT, aligns closely with established clinical markers of allergic sensitization, supporting its relevance and potential integration into the diagnostic workflow for AR.

## 4. Discussion

We developed a novel MAT system using CD63 as a core functional biomarker, demonstrating its utility as a complementary tool for the diagnosis of AR. In the field of allergological diagnosis, BAT and MAT are functional in vitro assays that utilize flow cytometry. Despite sharing a common principle of quantifying allergen‐induced degranulation by measuring the upregulation of surface activation markers (e.g., CD63 and CD203c), they are fundamentally distinct in terms of cell source, activation mechanisms, and clinical applications [15]. BAT primarily relies on fresh whole blood obtained from the patient, utilizing the patient’s own basophils to reflect IgE‐mediated allergic responses [10]. This is achieved by detecting the upregulation of surface activation markers, such as CD63. MAT is based on cultured mast cell lines, such as LAD2 or primary human mast cells. Its passive format, the pMAT, involves passive sensitization of donor mast cells with the patient’s serum or plasma, followed by allergen challenge to detect sIgE‐mediated degranulation [15, 16]. BAT is a relatively well‐established technique, supported by extensive clinical validation. It has demonstrated good sensitivity and specificity in diagnosing various IgE‐mediated allergies, including hymenoptera venom allergy, certain food allergies (e.g., to peanut and sesame), and immediate drug hypersensitivity reactions (e.g., to neuromuscular blocking agents) [17]. Consequently, it has been incorporated into the relevant clinical guidelines. A key advantage of the BAT is its capacity to reflect the “net effect” of a patient’s in vivo immune response [18]. This includes the complex interplay between cellular reactivity and humoral factors, making it particularly valuable for monitoring the efficacy of AIT. However, BAT is not without limitations. A significant drawback is the phenomenon of “nonresponder” basophils, which occurs in ~10%–15% of patients and renders the test results inconclusive. Furthermore, the sensitivity of the BAT can be suboptimal for allergens that elicit low levels of sIgE [19]. Regarding activation thresholds, BAT commonly uses an empirical criterion of >5% CD63‐positive basophils as an indicator of activation; however, this threshold is not universal and may vary depending on allergens, stimulation conditions, and analytical protocols [8]. Current recommendations suggest that allergen‐specific cutoffs should ideally be established using ROC analysis in well‐designed diagnostic studies. Similarly, the 2.09% CD63‐positive threshold established in our LAD2‐based MAT was derived from ROC analysis and represents an assay‐specific diagnostic criterion rather than a universal biological threshold. In comparison, research data on the MAT, while relatively recent and limited in scale, demonstrate promising diagnostic potential [11]. Specifically, passive MAT (pMAT) can provide reliable diagnostic information in cases where BAT is ineffective—such as in samples from “nonresponder” patients or those with low levels of sIgE [20, 21]. For example, in the context of peanut allergy discrimination, pMAT exhibits diagnostic performance comparable to, and in some cases superior to, that of BAT in differentiating clinically allergic patients from sensitized but tolerant individuals [9, 10]. Additionally, previous studies have shown that glucocorticoids can suppress IgE/FcεRI‐mediated activation of human basophils by affecting intracellular signaling pathways, which may potentially influence BAT results [22]. In our study, after excluding all participants with oral corticosteroid use, the differences in CD63 expression and histamine release between AR patients and controls remained consistent with the original analysis (Figure S2). However, the impact of medication exposure on MAT performance and its comparison with BAT require further investigation.

The MAT utilizing the LAD2 cell line can employ CD107a and CD107b as key activation markers in addition to CD63 [23]. The feasibility of this approach has been validated across multiple studies. CD107a (LAMP‐1) and CD107b (LAMP‐2) are lysosome‐associated membrane proteins that translocate to the cell surface during mast cell degranulation, with expression levels directly reflecting the degree of cellular activation [24]. Studies have confirmed strong positive correlations between CD63 and CD107 a/b expression in stimulated LAD2 cells (*r* = 0.70–0.89), supporting their synergistic use in monitoring degranulation dynamics [11]. Clinically, CD107a has demonstrated a high diagnostic performance. In a study using Hoxb8 mast cells—functionally analogous to LAD2—CD107a‐based MAT achieved 93% sensitivity and 96% specificity in peanut allergy diagnosis, matching the efficacy of CD63 while accurately identifying BAT nonresponders [25]. Moreover, combined detection of CD107a and CD107b improves the recognition of severe allergic reactions, with significantly higher activation levels observed in severe Hymenoptera venom allergy (Mueller grades III–IV) compared to mild–moderate cases. In summary, integrating CD107a and CD107b into LAD2‐based MAT protocols enhances test reliability through multiparameter analysis and provides a more comprehensive profile of mast cell activation [11]. This approach represents a promising advancement in functional allergy diagnostics.

MAT serves as a robust functional in vitro assay for detecting IgE‐mediated mast cell degranulation, enabling precise diagnosis of allergic conditions. In food allergy, MAT demonstrates superior discriminatory power over skin prick tests and specific IgE assays. For peanut allergy, it achieves sensitivity and specificity comparable to the BAT while effectively overcoming BAT’s “nonresponder” limitation, accurately distinguishing clinically allergic patients from sensitized but tolerant individuals [25, 26]. MAT shows significant utility in drug hypersensitivity studies, reliably identifying IgE‐mediated reactions, as validated in chlorhexidine allergy models [27, 28]. It also contributes to chronic spontaneous urticaria (CSU) research by detecting functional autoantibodies against FcεRI/IgE in patient serum, facilitating endotyping and biomarker development [20, 29]. In tracking treatment responses, MAT dynamically captures cellular‐level changes following AIT or biologic treatments (e.g., omalizumab). By monitoring reductions in mast cell reactivity, it emerges as a promising biomarker for evaluating efficacy and predicting immune tolerance induction.

Beyond the diagnostic performance, the translational feasibility of LAD2‐based MAT requires further consideration. MAT offers clear practical advantages over BAT: it avoids fresh blood requirements, minimizes donor variability via a standardized, cryopreservable cell source, improves reproducibility, allows flexible batch testing without immediate processing, and requires minimal serum. However, LAD2 cells grow slowly and demand specialized media, SCF supplementation, and strict quality control, increasing complexity and costs compared with automated, cost‐effective ELISA/ImmunoCAP platforms. While ELISA/ImmunoCAP only reflects sensitization, MAT provides functional mast cell degranulation data, offering clinical value in cases with discordant serology and symptoms. Future efforts should optimize culture protocols (e.g., reducing culture duration, minimizing cell numbers, and lowering costs) to improve scalability and establish MAT as a complementary functional diagnostic tool in specialized allergy laboratories.

Several limitations of the present study should be acknowledged. Although the case and control groups were not fully matched for age and sex, all participants were within the same age range (18–65 years). Previous studies have shown that allergen‐specific IgE levels and allergic symptom severity generally decrease with age [30]. The older control group (39.20 ± 12.70 vs. 29.60 ± 15.50 years) may have lower IgE production and allergic reactivity, potentially overestimating the disease‐associated effect. Conversely, estrogen can enhance IgE‐dependent mast cell activation [31]. The control group had more females (67.8% vs. 54.7%), which may increase mast cell reactivity in controls and underestimate between‐group differences. Thus, the potential confounding effects of age and sex may operate in opposite directions. Future studies with age‐ and sex‐matched cohorts or multivariable adjustment for age and sex are needed to determine the independent effect of AR and validate these findings. Additionally, the moderate correlation between CD63 and sIgE, together with discordant cases (high sIgE with low CD63 responses or relatively low sIgE with high CD63 responses), indicates that allergen sensitization and functional mast cell activation are related but not equivalent biological processes. Serum sIgE reflects the presence and level of allergen‐specific antibodies, whereas MAT evaluates the downstream IgE/FcεRI‐mediated mast cell activation. High sIgE levels do not always correlate with effector cell activation due to differences in IgE functional affinity, the ratio of allergen‐specific to tIgE, and mast cell reactivity; low‐affinity IgE or limited epitopes may fail to effectively crosslink FcεRI [32]. Furthermore, the differences in the composition of allergen components and the recognition of epitopes may affect functional activation [33]. Since our controls were healthy rather than asymptomatically sensitized, validation in a sensitized but clinically tolerant/asymptomatic cohort is needed to determine whether MAT can distinguish symptomatic allergy from asymptomatic sensitization.

## 5. Conclusions

The present study established a CD63‐based MAT as a novel functional assay for the in vitro diagnosis of AR. This MAT system demonstrated potential as a reliable functional allergy diagnostic tool with promising clinical applicability, serving as a valuable complement to existing serological testing methodologies.

## Funding

This work was supported by the National Natural Science Foundation of China (Grants 81903572 and U24A20786), the Natural Science Foundation of Shaanxi Province (Grant 2022JQ‐858), and the Basic Research Funds for Central Universities (Grant xzy012024150).

## Conflicts of Interest

The authors declare no conflicts of interest.

## Supporting Information

Additional supporting information can be found online in the Supporting Information section.

## Supporting information


**Supporting Information** Supporting figures and table related to the mast cell activation test, including the flow cytometric gating strategy for LAD2 cells (Figure S1), sensitivity analysis after excluding participants with oral corticosteroid use (Figure S2), and quality control parameters of LAD2 cells across consecutive batches (Table S1).

## Data Availability

The data supporting the findings of this study are available from the corresponding author upon reasonable request.
