# Supplementary material for: Development of a Mast‐Cell Activation Test for Diagnosing Allergic Rhinitis
Source: J Immunol Res. 2026 Nov 9;2026:6317442. doi: 10.1155/jimr/6317442 (PMC13749072; doi:10.1155/jimr/6317442)
Supplement: Supplementary file 1 [file JIMR-2026-6317442-s001.docx]

**Figure S1. Complete gating strategy for flow cytometric analysis of LAD2 mast cell activation.** Representative flow cytometry plots showing the sequential gating strategy used for analysis. Cells were first identified based on forward scatter (FSC) and side scatter (SSC) characteristics, followed by singlet discrimination using FSC-A/FSC-H. Live cells were then selected, and CD63-positive mast cells were quantified within the live cell population.

**Figure S2. Sensitivity analysis of mast cell activation markers after excluding participants with oral corticosteroid use.** (A) CD63⁺ expression levels in allergen-stimulated mast cells from AR cases and healthy controls after exclusion of all participants with oral corticosteroid use (case: n = 77; control: n = 61; ^***^*P* < 0.001 vs. control group). (B) Histamine release from allergen-stimulated mast cells in AR cases and healthy controls after exclusion of all participants with oral corticosteroid use (case: n = 77; control: n = 61; ^***^*P* < 0.001 vs. control group). Data are presented as mean ± SEM. Statistical significance was determined by the Mann–Whitney U test.

**Table S1. Quality control parameters of LAD2 cells across consecutive batches used for clinical sample testing**

| Batch | Baseline CD63 (%) | Positive Control CD63 (%) |
| --- | --- | --- |
| Batch 1 | 0.72 | 74.6 |
| Batch 2 | 0.67 | 82.9 |
| Batch 3 | 0.45 | 77.3 |
| Batch 4 | 0.71 | 70.3 |
| Batch 5 | 0.40 | 73.40 |
| Mean ± SD | 0.59 ± 0.15 | 75.70 ± 4.75 |
| CV (%) | 25.90% | 6.27% |
